# Dysregulation of PI4P in the *trans* Golgi regions activates the mammalian Golgi stress response

**DOI:** 10.1016/j.jbc.2024.108075

**Published:** 2024-12-13

**Authors:** Kanae Sasaki, Marika Toide, Takuya Adachi, Fumi Morishita, Yuto Watanabe, Hajime Tajima Sakurai, Sadao Wakabayashi, Satoshi Kusumi, Toshiyuki Yamaji, Kaori Sakurai, Daisuke Koga, Kentaro Hanada, Masafumi Yohda, Hiderou Yoshida

**Affiliations:** 1 Department of Molecular Biochemistry, Graduate School of Science, University of Hyogo, Ako, Hyogo, Japan; 2 Division of Morphological Sciences, Kagoshima University Graduate School of Medicine and Dental Sciences, Kagoshima, Kagoshima, Japan; 3 Department of Biochemistry and Cell Biology, National Institute of Infectious Diseases, Shinjuku, Tokyo, Japan; 4 Faculty of Pharmacy, Department of Microbiology and Immunology, Juntendo University, Urayasu, Chiba, Japan; 5 Faculty of Engineering, Department of Biotechnology and Life Science, Tokyo University of Agriculture and Technology, Koganei, Tokyo, Japan; 6 Department of Microscopic Anatomy and Cell Biology, Asahikawa Medical University, Asahikawa, Hokkaido, Japan; 7 Center for Quality Management Systems, National Institute of Infectious Diseases, Shinjuku, Tokyo, Japan

**Keywords:** Golgi stress response, phosphatidylinositol-4-phosphate, the genome-wide CRISPR-Cas9 KO screening, OSW-1, cancer

## Abstract

The Golgi stress response is an important cytoprotective system that enhances Golgi function in response to cellular demand, while cells damaged by prolonged Golgi stress undergo cell death. OSW-1, a natural compound with anticancer activity, potently inhibits OSBP that transports cholesterol and phosphatidylinositol-4-phosphate (PI4P) at contact sites between the endoplasmic reticulum and the Golgi apparatus. Previously, we reported that OSW-1 induces the Golgi stress response, resulting in Golgi stress-induced transcription and cell death. However, the underlying molecular mechanism has been unknown. To reveal the mechanism of a novel pathway of the Golgi stress response regulating transcriptional induction and cell death (the PI4P pathway), we performed a genome-wide KO screen and found that transcriptional induction as well as cell death induced by OSW-1 was repressed by the loss of regulators of PI4P synthesis, such as PITPNB and PI4KB. Our data indicate that OSW-1 induces Golgi stress-dependent transcriptional induction and cell death through dysregulation of the PI4P metabolism in the Golgi.

Each organelle in eukaryotic cells has its own mechanism of the stress response that maintains homeostasis of the organelle to cope with insufficiency of organelle function. For example, the endoplasmic reticulum (ER), where membrane and secretory proteins are synthesized and properly folded, has the mechanism of the stress response called the ER stress response (also called the unfolded protein response) (1, 2, 3, 4, 5, 6). The mammalian ER stress response consists of three response pathways: the IRE1, ATF6, and PERK pathways, which upregulate the expression of ER chaperones and ER-associated degradation factors and repress translation upon accumulation of unfolded or misfolded proteins in the ER.

The Golgi apparatus is an organelle that executes posttranslational modifications including glycosylation and sorting of membrane proteins and secretory proteins. When the production of these proteins in the ER is increased and a majority of them are transported into the Golgi, the function of the Golgi becomes insufficient (Golgi stress). To augment the function and relieve Golgi stress, cells activate the Golgi stress response to upregulate the expression of Golgi-related proteins such as glycosylation enzymes and transport components at the level of transcription (7). Previously, we identified three response pathways controlling the Golgi stress response: the TFE3 pathway (8), the proteoglycan pathway (9) and the mucin pathway (10), which augment general function of the Golgi, proteoglycan-type glycosylation and mucin-type glycosylation, respectively.

OSW-1 is a natural anticancer compound isolated from the bulbs of *Ornithogalum saundersiae*, which is a herbaceous plant belonging to Liliaceae family (11, 12). OSW-1 has been shown to inhibit cell proliferation and induce apoptosis in cancer cells with high selectivity (12). However, the mechanism by which OSW-1 induces cell death has been controversial. Several research groups have reported that OSW-1 depolarizes mitochondrial membranes and induces mitochondria-dependent apoptosis with a rise in cytoplasmic calcium (13), for example, by inhibition of sodium-calcium exchanger 1 (14). Other researchers have reported mitochondria-independent apoptosis by OSW-1 (15, 16).

Oxysterol-binding protein (OSBP) and OSBP2 belong to a family of lipid transfer proteins highly conserved in eukaryotes (the OSBP-related protein family) and act as transporters of lipids such as cholesterol and phospholipids at membrane contact sites (MCSs) (17). OSBP contains a pleckstrin homology (PH) domain, FFAT-motif and OSBP-related domain (ORD). OSBP is localized to MCSs between the ER and the Golgi through its PH domain interacting with phosphatidylinositol-4-phosphate (PI4P) and the small GTPase ARF1 at the Golgi membranes and the FFAT-motif interacting with VAMP-associated protein A/B (VAPA/B) at the ER membranes (17). OSBP transfers PI4P from the Golgi to the ER, while it transports cholesterol from the ER to the Golgi. This PI4P consumption at the Golgi membranes enables OSBP to drive the delivery of cholesterol from the ER (18). Interestingly, OSBP and OSBP2 were identified as molecular targets of OSW-1 (19). OSW-1 inhibits the exchange transport of cholesterol and PI4P between the ER and the Golgi (20). OSBP2 is the protein most closely related to OSBP and also has a PH domain, FFAT-motif and ORD, as well as OSBP. OSBP2 forms a heterodimer with OSBP and is localized to the Golgi (21), though the function of OSBP2 has not been clarified yet in terms of the lipid transfer at MCSs between the ER and the Golgi.

We have previously discovered that OSW-1 activates the TFE3 pathway of the Golgi stress response in HeLa cells (22), while Oh-hashi et al., have recently reported that OSW-1 induces atypical Golgi stress and autophagy in Neuro2a cells (23). Therefore, we speculated that the Golgi stress response also contributes to OSW-1-induced transcription and cell death in addition to mitochondrial depolarization. In this report, to clarify the primary cause of cell death induced by OSW-1, we searched for genes essential for the cell death using the genome-wide CRISPR-Cas9 knockout (GeCKO) system.

## Results

### Inhibition of OSBP and OSBP2 by OSW-1 induces transcription of OSBP2 through the PI4P pathway of the Golgi stress response

**Figure 1:**
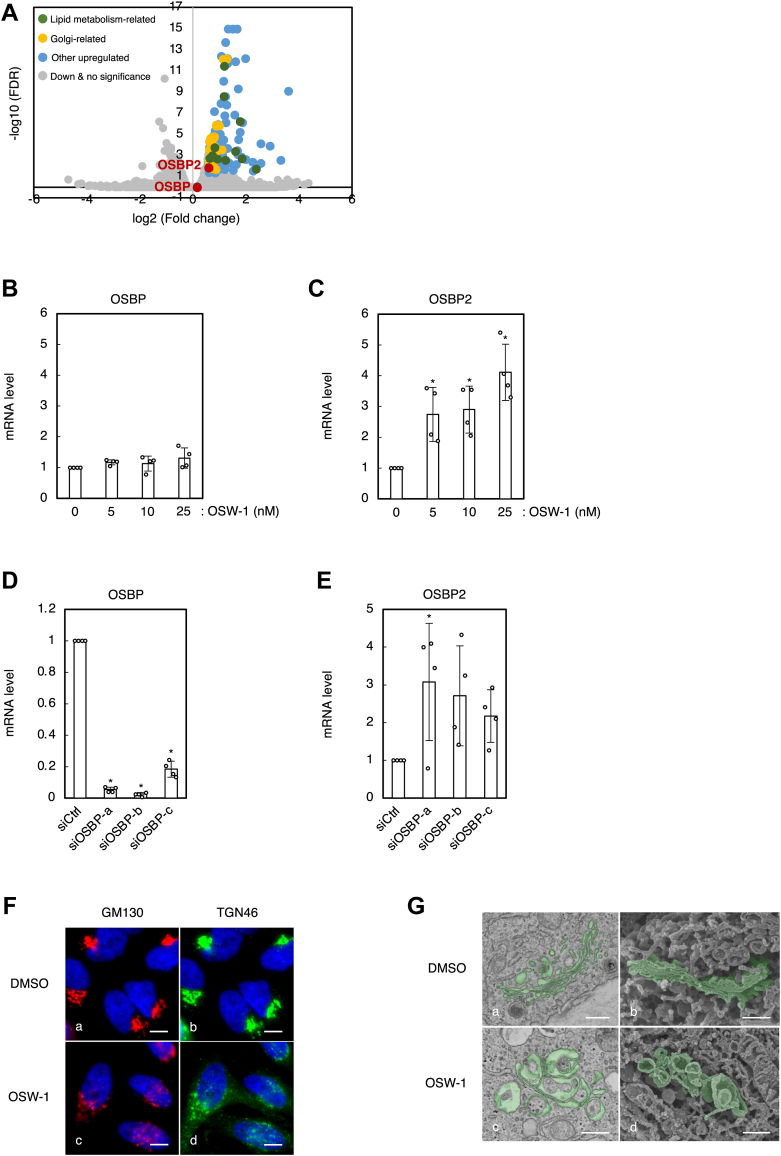
**The effect of OSW-1 treatment on transcription and the morphology of the Golgi**. *A*, volcano plot showing differentially expressed genes in HeLa cells between groups treated with or without OSW-1. Total RNA extracted from HeLa cells treated with or without 5 nM OSW-1 for 18 h was subjected to RNA-seq analysis. *Green* (lipid metabolism-related), *yellow* (Golgi-related), and *blue* (other) dots represent significantly upregulated genes (FDR<0.05, Fold change>1.5). *Gray dots* represent downregulated and insignificantly differential expressed genes. *B*–*E*, quantitative real-time PCR (qRT-PCR) analysis of OSBP and OSBP2 mRNA. Total RNA extracted from HeLa cells treated with or without OSW-1 (*B* and *C*) or OSBP-knockdown cells (*D* and *E*) was subjected to qRT-PCR. All mRNA levels were normalized to GAPDH mRNA, which was used as a reference. ∗*p* < 0.05 *versus* each control (analysis of variance followed by Dunnett’s test; mean ± S.D.; n = 4). *F*, immunofluorescence microscopic analysis of the Golgi. Wt HeLa cells were treated with or without 5 nM OSW-1 for 18 h and stained with murine anti-GM130 mAb (*red*, *A* and *C*), sheep anti-TGN46 pAb (*green*, *B* and *D*), and with DAPI (*blue*). The scale bars represent 10 μm. *G*, electron microscopy analysis of the Golgi. HeLa cells were treated without (*A* and *B*) or with 5 nM OSW-1 (*C* and *D*) for 18 h, and subjected to SEM analysis. The scale bars represent 500 nm. DAPI, 4′,6-diamidino-2-phenylindole; OSBP, oxysterol-binding protein; pAb, polyclonal antibody; RNA-seq, RNA sequencing; SEM, scanning electron microscopy; TGN, *trans* Golgi network.

OSW-1 binds to the ORD of OSBP and OSBP2, and inhibits cholesterol transfer from the ER to the Golgi and PI4P transfer from the Golgi to the ER (19, 20). We previously found that the inhibition of OSBP and OSBP2 by OSW-1 increases the transcription of target genes of the TFE3 pathway of the Golgi stress response such as GM130 and GCP60 (22). To get a comprehensive view of the transcriptome of cells treated with OSW-1, we performed RNA sequencing (RNA-seq) with next-generation DNA sequencing technology. Total RNA was prepared from HeLa cells treated with OSW-1 and subjected to RNA-seq (the complete results of RNA-seq were shown in Datas S1 and S2). As shown in Fig. 1*A*, expression of genes involved in Golgi function and lipid metabolism was upregulated upon OSW-1 treatment (Fig. 1*A*, Tables 1 and 2). Interestingly, we found that these upregulated genes included the OSBP2 gene, the transcription of which was increased significantly upon OSW-1 treatment (Fig. 1*A* and Table 2). In contrast, transcription of OSBP was hardly affected (Fig. 1*A*) although both OSBP and OSBP2 are targets of inhibition by OSW-1. We performed quantitative real time PCR (qRT-PCR) analysis using the same RNA samples, and obtained the results similar with those from the RNA-seq (Fig. 1, *B* and *C*). Suppression of OSBP expression with siRNA of OSBP (siOSBP) also resulted in the increase of OSBP2 mRNA, indicating that inhibition of OSBP and OSBP2 by OSW-1 induces transcription of OSBP2 (Fig. 1, *D* and *E*). Since OSBP2 is not a target gene of the TFE3 pathway (24), another response pathway of the Golgi stress response induces transcription of OSBP2. We named this novel response pathway the PI4P pathway.

**Table 1:** Golgi-related genes whose expression is induced by OSW-1

Gene	Protein	Fold change	FDR
KDELR3	ER lumen protein-retaining receptor 3	2.43	8.31E-13
SEC24D	Protein transport protein Sec24D	2.22	8.36E-13
GALNT3	Polypeptide N-acetylgalactosaminyltransferase 3	2.15	0.000308
COG6	Conserved oligomeric Golgi complex subunit 6	1.99	1.66E-06
GOLGA2	Golgin subfamily A member 2	1.85	1.36E-06
ARFGAP3	ADP-ribosylation factor GTPase-activating	1.84	1.85E-06
FAM18B2	Golgi apparatus membrane protein TVP23 homolog C	1.83	0.00282
TMF1	TATA element modulatory factor	1.82	0.0233
GNPTAB	N-acetylglucosamine-1-phosphotransferase subunits alpha/beta	1.80	0.000618
SEC24A	Protein transport protein Sec24A	1.77	0.000145
IFT20	Intraflagellar transport protein 20 homolog	1.75	1.88E-05
FAM18B1	Golgi apparatus membrane protein TVP23 homolog B	1.74	0.000381
SEC13	Protein SEC13 homolog	1.74	2.44E-05
RAB9A	Ras-related protein Rab-9A	1.72	0.0192
CHPF2	Chondroitin sulfate glucuronyltransferase	1.70	0.000145
KDELR2	ER lumen protein-retaining receptor 2	1.69	1.84E-05
YIPF2	Protein YIPF2	1.68	0.00866
OPTN	Optineurin	1.67	0.000827
CLINT1	Clathrin interactor 1	1.66	0.00380
ST3GAL1	CMP-N-acetylneuraminate-beta-galactosamide-alpha-2,3-sialyltransferase 1	1.64	0.0148
COPB2	Coatomer subunit beta'	1.63	0.000211
LMAN1	Protein ERGIC-53	1.63	0.000602
CTAGE5	Melanoma inhibitory activity protein 2	1.62	0.00242
ARFGAP1	ADP-ribosylation factor GTPase-activating protein 1	1.60	0.0183
CHPF	Chondroitin sulfate synthase 2	1.60	0.000363
COPB1	Coatomer subunit beta	1.59	2.52E-05
ARL1	ADP-ribosylation factor-like protein 1	1.58	0.000224
SEC31 A	Protein transport protein Sec31 A	1.58	0.0165
ACBD3	Golgi resident protein GCP60	1.57	0.000935
YIF1A	Protein YIF1A	1.56	0.00304
SAR1A	GTP-binding protein SAR1a	1.56	0.00566
YIPF5	Protein YIPF5	1.54	5.41E-05
BET1L	BET1-like protein	1.54	0.00933
TMED7	Transmembrane emp24 domain-containing protein 7	1.53	0.000322
COPA	Coatomer subunit alpha	1.53	0.0109
SLC35A2	UDP-galactose translocator	1.53	0.00933
SCYL1	N-terminal kinase-like protein	1.52	0.0101
B4GALT5	Beta-1,4-galactosyltransferase 5	1.52	0.00596
COPG1	Coatomer subunit gamma-1	1.52	0.00264
EXT1	Exostosin-1	1.51	0.0148

RNA sequencing was performed using total RNA extracted from HeLa cells treated with or without 5 nM OSW-1 for 18 h. Golgi-related genes whose expression was induced more than 1.5-fold in OSW-1-treated cells compared with untreated control cells are listed. FDR, false discovery rate.

**Table 2:** Lipid metabolism-related genes whose expression is induced by OSW-1

Gene	Protein	Fold change	FDR
NR5A2	Nuclear receptor subfamily 5 group A member 2	5.22	0.0210
MALL	MAL-like protein	3.59	0.00220
FABP3	Fatty acid-binding protein, heart	3.43	6.32E-07
ATP8B1	Phospholipid-transporting ATPase IC	3.05	0.000424
RORC	Nuclear receptor ROR-gamma	2.34	0.00296
ARID5B	AT-rich interactive domain-containing protein 5B	2.27	3.87E-12
PPAPDC1B	Phospholipid phosphatase 5	2.26	2.82E+00
AGPAT2	1-acyl-sn-glycerol-3-phosphate acyltransferase beta	1.90	0.00231
TMEM39A	Transmembrane protein 39A	1.77	0.000199
PITPNC1	Cytoplasmic phosphatidylinositol transfer protein 1	1.66	0.00107
LPCAT1	Lysophosphatidylcholine acyltransferase 1	1.63	0.00174
ATP8B2	Phospholipid-transporting ATPase ID	1.56	0.00191
OSBP2	Oxysterol-binding protein 2	1.51	0.0145

RNA sequencing was performed using total RNA extracted from HeLa cells treated with or without 5 nM OSW-1 for 18 h. Lipid metabolism-related genes whose expression was induced more than 1.5-fold in OSW-1-treated cells compared with untreated control cells are listed. FDR, false discovery rate.

Previously, it was reported that OSW-1 treatment induces fragmentation of the Golgi (19). As shown in Figure 1*F*, we also confirmed Golgi fragmentation by OSW-1 with immunofluorescence analysis using anti-GM130 and anti-TGN46 antibodies in HeLa cells (panels c and d). To precisely analyze microstructural changes of the Golgi by OSW-1, we performed scanning electron microscopy (SEM) of HeLa cells treated with OSW-1 for 18 h (Fig. 1*G*). Each Golgi cisterna was flat and orderly in HeLa cells without OSW-1 treatment (panels a and b), whereas cells treated with OSW-1 showed swelling and vacuolation of most cisternae (panels c and d). We confirmed that these swollen membrane structures were originated from cisternae of the Golgi using the immunoelectron microscopy method (Fig. S1). As shown in Figure 1*F* (panels a and b), a set of the Golgi stack was positioned near the nucleus under the normal condition. On the other hand, there were several fragmented Golgi stacks and many vacuoles in OSW-1-treated cells (panels c and d).

### Screening of genes involved in cell death induced by OSW-1

**Figure 2:**
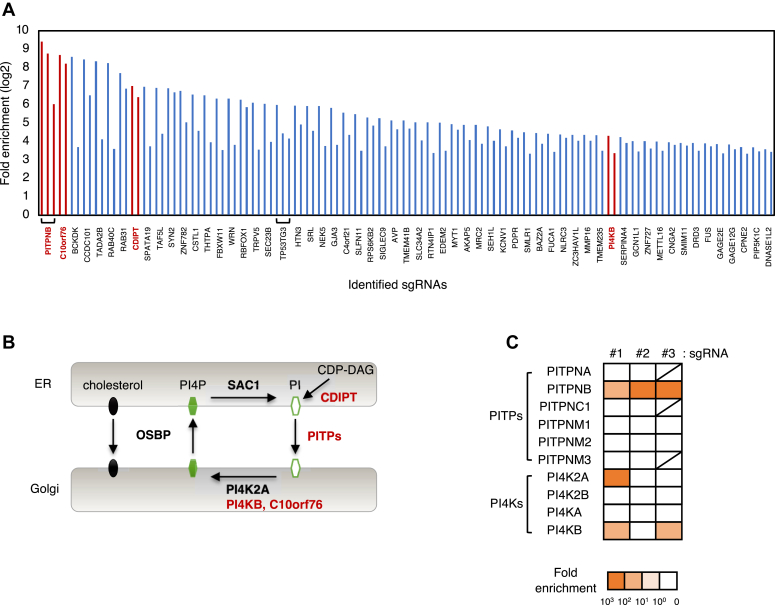
**Identification of genes involved in cell death induced by OSW-1 using GeCKO screening**. *A*, identification of sgRNAs enriched in the OSW-1-resistant HeLa cells. Fold enrichment corresponds to the average of two independent experiments. *Red bars* show three sgRNAs targeting the PITPNB gene, two sgRNAs targeting the C10orf76 gene, 2 sgRNAs targeting the CDIPT gene, and 2 sgRNAs targeting the PI4KB gene. *B*, a schematic presentation of PI4P metabolism in the ER and the Golgi. Genes enriched in the screening are shown in *red*. *C*, heatmap showing fold enrichment of individual sgRNA (#1–3) targeting genes of PITPs and PI4Ks. The *shaded lines* mean that the third sgRNAs (#3) targeting PITPNA, PITPNC1, and PITPNM3 are not present. ER, endoplasmic reticulum; GeCKO, genome-wide CRISPR-Cas9 knockout; PI4K, PI 4-kinase; PI4P, phosphatidylinositol-4-phosphate; PITP, (PI)-transfer protein; sgRNA, single guide RNA.

It was previously reported that OSW-1 induces cell death (12), and that the Golgi stress response is involved in OSW-1-induced cell death (22, 23). However, it has been elusive how the Golgi stress response induces cell death. To identify genes responsible for the OSW-1-induced cell death, we performed GeCKO screening using a GeCKO v2 pooled library A. Briefly, 60 genes (corresponding to 122 single guide RNAs (sgRNAs)) were extracted as the candidates, each of which included multiple sgRNAs enriched in the range of 10- to 681-fold (Fig. 2*A*). Among them, we focused on four genes: PITPNB, C10orf76/DGARM/ARMH3, CDIPT, and PI4KB, which are related to PI4P synthesis in the Golgi (Fig. 2*B*). PITPNB is a member of the phosphatidylinositol (PI)-transfer protein (PITP) family, which catalyzes the transfer of PI from the ER to the Golgi (25, 26). The sgRNAs targeted to other members of PITPs (PITPNA, PITPNC1, PITPNM1, PITPNM2, and PITPNM3) were not enriched in this screening (Fig. 2*C*). CDIPT is CDP-diacylglycerol-inositol 3-phosphatidyl transferase, which catalyzes the *de novo* synthesis of PI from CDP-diacylglycerol (CDP-DAG) (27, 28). PI4KB is one of PI 4-kinases (PI4Ks), which produce PI4P from PI, and mainly localized in the *trans* Golgi stacks and the *trans* Golgi network (TGN) (hereafter we use the term “*trans*-Golgi regions” to represent the *trans* Golgi stacks and TGN) (29, 30). Four PI4Ks (PI4K2A, PI4K2B, PI4KA, and PI4KB) exist in mammalian cells, and only one type of sgRNA targeting PI4K2A was enriched in addition to multiple sgRNAs targeting PI4KB (Fig. 2*C*). PI4K2A is also responsible for PI4P production in the *trans* Golgi regions (31). DGARM is involved in the activation of PI4KB (32, 33). This result suggests that PI4P metabolism in the *trans* Golgi regions is closely related to OSW-1-induced cell death.

### Either PITPNB or PI4KB gene is indispensable for cell death induced by OSW-1

**Figure 3:**
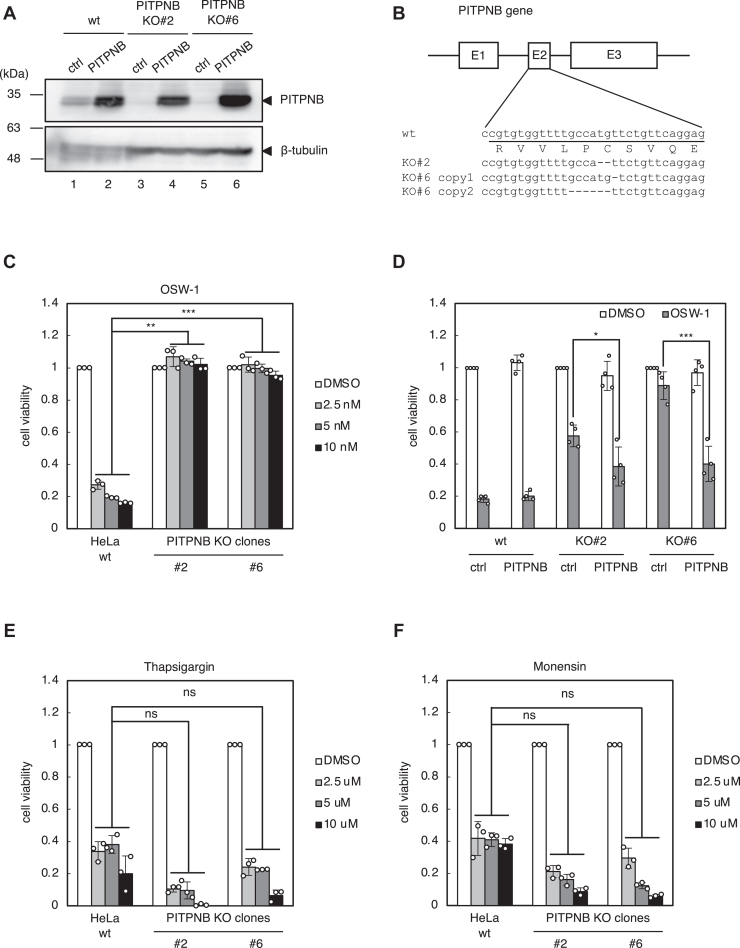
**OSW-1-induced cell death in PITPNB KO cells**. *A*, immunoblot analysis of PITPNB protein. Wt HeLa cells and two individual clones (#2 and #6) of PITPNB KO cells were transfected with an empty vector (ctrl) or an expression plasmid of PITPNB (PITPNB). β-tubulin was used as a loading control. *B*, genomic sequences of exon 2 of human PITPNB gene in wt HeLa cells and PITPNB KO clones (#2 and #6). Deletion of two nucleotides was confirmed in both copies of the PITPNB gene in KO#2, resulting in a frameshift. Deletion of one nucleotide or six nucleotides was confirmed in each copy of the PITPNB gene in KO# 6. *C*–*F*, cell viability assay. Wt HeLa cells and PITPNB KO clones (#2 and #6) were treated with or without the indicated concentrations of OSW-1, thapsigargin, or monensin for 48 h, and subjected to cell viability assay. ∗∗*p* < 0.01, ∗∗∗*p* < 0.001 *versus* wt cells supplemented with the same concentration of each reagent (the Bonferroni-corrected *t* test; mean ± S.D.; n = 3). ns, not significant. *D*, rescue experiments of PITPNB KO cells. Wt HeLa cells and PITPNB KO clones (#2 and #6) were transfected with an empty vector (ctrl) or an expression plasmid of PITPNB (PITPNB), treated with or without 5 nM OSW-1 for 48 h, and subjected to cell viability assay. ∗*p* < 0.05, ∗∗∗*p* < 0.001 (Student’s *t* test; mean ± S.D.; n = 4). PITP, (PI)-transfer protein.

The GeCKO v2 pooled library A contains three sgRNAs targeting PITPNB, all of which were highly enriched in the screening (Fig. 2*A*), suggesting that PITPNB is the most promising candidate. We established two stable cell lines of PITPNB KO cells (PITPNB KO#2 and #6). To check the expression of PITPNB protein in these clones, we made immunoblotting with anti-PITPNB antibody (Fig. 3*A*). Although the protein levels of PITPNB in PITPNB KO#2 and KO#6 cells were remarkably reduced compared to wt HeLa cells, we detected faint bands at the same position as those of PITPNB proteins in wt cells (lanes 3 and 5). To confirm that the PITPNB gene was completely disrupted in PITPNB KO#2 and KO#6 cells, we checked the nucleotide sequences of the second exon of the PITPNB gene in these cells, which contains a target sequence of an sgRNA used to establish PITPNB KO cell lines (Fig. 3*B*). PITPNB KO#2 showed the deletion of two sequential nucleotides (2 nt) upstream to the sgRNA target region, which caused a frameshift of translation and loss of its mature protein. PITPNB KO#6 showed two patterns of genome sequences: one was 1 nt deletion resulting in a frameshift, and the other was 6 nt deletion, which did not cause a frameshift. Although these results could not exclude the possibility that each clone had at least one copy with no editing or no frameshift, cell death by OSW-1 was markedly repressed in PITPNB KO#2 and KO#6 compared to wt cells (Fig. 3*C*). Importantly, the ectopic expression of PITPNB (Fig. 3*A*, lanes 4 and 6) rescued the sensitivity to OSW-1 in PITPNB KO cells (Fig. 3*D*), indicating that the resistance to OSW-1 is ascribed to the loss of PITPNB, not to any off-targeting effects of gene disruption experiments. To rule out the possibility that the cell death machinery itself is lost in PITPNB KO#2 and KO#6 cells, we examined the sensitivity of PITPNB KO#2 and KO#6 cells to other stress inducers (Fig. 3, *E* and *F*). Thapsigargin, an inducer of the ER stress, killed PITPNB KO#2 and KO#6 cells at the same sensitivity as wt cells, verifying that the cell death machinery itself is intact in PITPNB KO#2 and KO#6 cells. Sensitivity to monensin, an activator of the TFE3 pathway of the Golgi stress response, was the same for both wt and PITPNB KO cells. This suggested that the PI4P pathway activated by OSW-1 is distinct from the TFE3 pathway.

**Figure 4:**
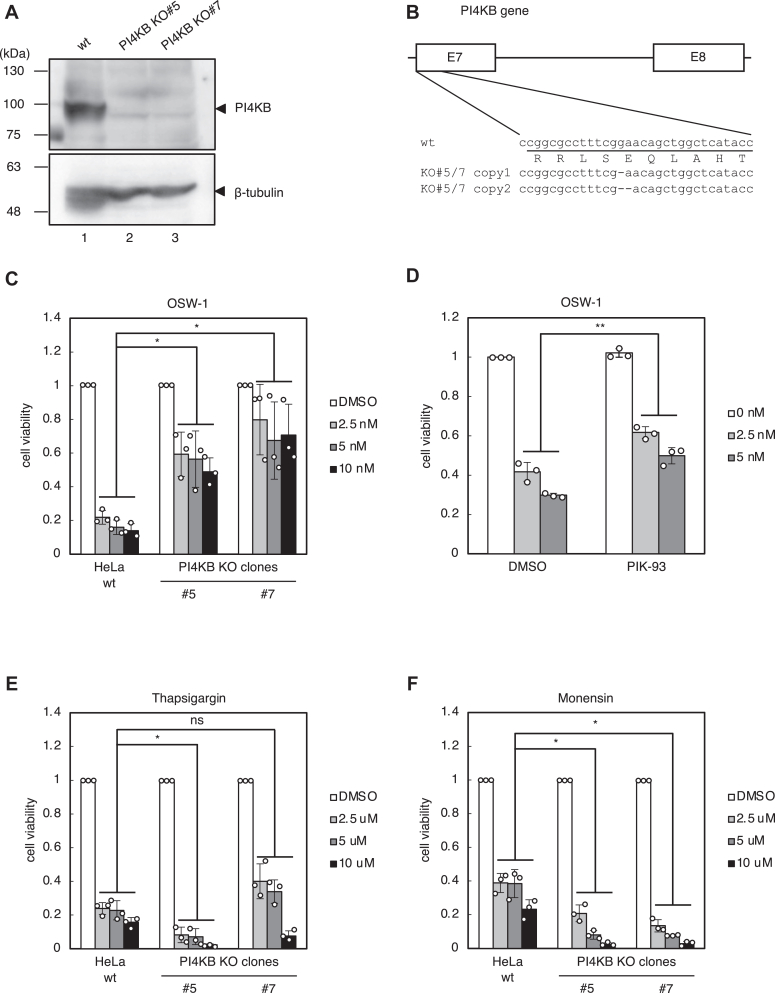
**OSW-1-induced cell death in PI4KB KO cells**. *A*, immunoblot analysis of PI4KB protein in wt HeLa cells and two individual clones (#5 and #7) of PI4KB KO cells. β-tubulin was used as a loading control. *B*, genomic sequences of exon 7 of human PI4KB gene in wt HeLa cells and PI4KB KO clones (#5 and #7). Deletion of one nucleotide or two nucleotides was confirmed in each copy of the PI4KB gene in KO#5 and KO#7, resulting in a frameshift. *C*, cell viability assay. Wt HeLa cells and PI4KB KO clones (#5 and #7) were treated with or without the indicated concentrations of OSW-1 for 48 h, and subjected to cell viability assay. ∗*p* < 0.05 *versus* wt cells supplemented with the same concentration of each reagent (the Bonferroni-corrected *t* test; mean ± S.D.; n = 3). ns, not significant. *D*, cell viability assay. Wt HeLa cells were pretreated for 30 min with 500 nM PIK-93. Then the cells were treated with or without OSW-1 (2.5 nM or 5 nM) for 48 h without removal of PIK-93, and subjected to cell viability assay. ∗∗*p* < 0.01 *versus* wt cells supplemented with the same concentration of each reagent (Student’s *t* test; mean ± S.D.; n = 3). ns, not significant. *E* and *F*, cell viability assay. Wt HeLa cells and PI4KB KO clones (#5 and #7) were treated with or without the indicated concentrations of thapsigargin or monensin for 48 h, and subjected to cell viability assay. ∗*p* < 0.05 *versus* wt cells supplemented with the same concentration of each reagent (the Bonferroni-corrected *t* test; mean ± S.D.; n = 3). ns, not significant; PI4K, PI 4-kinase

Two stable cell lines of PI4KB KO cells (PI4KB KO #5 and KO#7) were also established. Immunoblotting analysis showed that the expression of PI4KB protein was completely lost (Fig. 4*A*, lanes 2 and 3). Genomic DNA analysis revealed that both alleles of the PI4KB gene were disrupted with 1 and 2 nt deletions, which cause frameshift (Fig. 4*B*). Cell death by OSW-1 was suppressed in PI4KB KO #5 and KO#7 cells compared to wt cells (Fig. 4*C*). To confirm whether the enzymatic activity of PI4KB is involved in OSW-1-induced cell death, we used PIK-93, which is the specific inhibitor of PI4KB (18). The treatment of PIK-93 mitigated OSW-1-induced cell death as well as KO of PI4KB (Fig. 4*D*). As an alternative approach, we tried to overexpress SAC1, which is a phosphatase of PI4P localized in the ER. Although we used both SAC1 wt and its Golgi-localized mutant K2A, their overexpression did not suppress cell death by OSW-1 (Fig. S2). Overexpression of SAC1 in our experiments may not be sufficient to reduce PI4P levels in the *trans* Golgi regions effectively. The cell death machinery was not disrupted in PI4KB KO #5 and KO#7 cells since sensitivity to thapsigargin and monensin is the same for PI4KB KO #5, KO#7 and wt cells (Fig. 4, *E* and *F*). These results lead us to the previously unrecognized possibility that PI4P levels in the *trans* Golgi regions may be responsible for cell death caused by OSW-1.

### Either PITPNB or PI4KB is indispensable for the transcriptional induction of target genes of the PI4P pathway in response to Golgi stress

**Figure 5:**
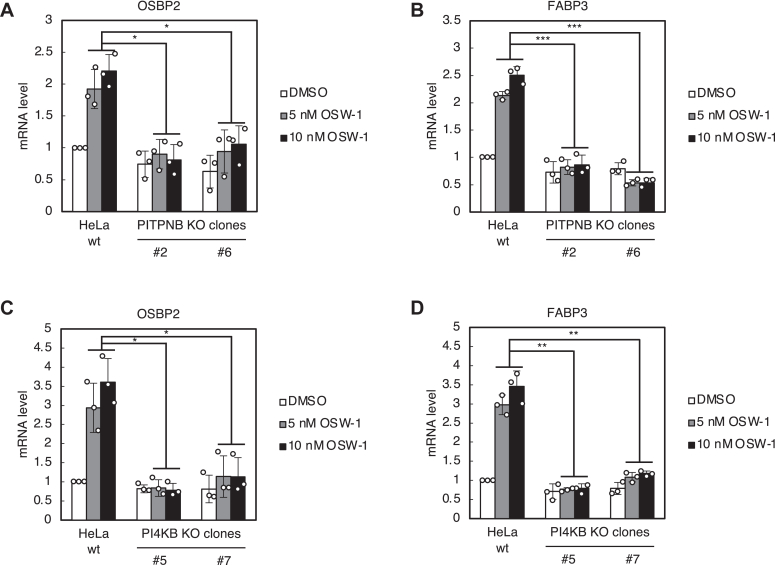
**Transcriptional induction of OSBP2 and FABP3 genes by OSW-1 in PITPNB KO and PI4KB KO cells**. *A*–*D*, qRT-PCR analysis of OSBP2 and FABP3 mRNA. Wt HeLa cells, PITPNB KO clones (#2 and #6), and PI4KB KO clones (#5 and #7) were treated with or without 5 nM OSW-1 for 18 h, and subjected to qRT-PCR analysis. All mRNA levels were normalized to GAPDH mRNA, which was used as a reference. ∗*p* < 0.05, ∗∗*p* < 0.01, ∗∗∗*p* < 0.001 (the Bonferroni-corrected *t* test; mean ± S.D.; n = 3). OSBP, oxysterol-binding protein; PITP, (PI)-transfer protein; PI4K, PI 4-kinase; qRT-PCR, quantitative real time PCR.

RNA-seq on HeLa cells treated with OSW-1 revealed the transcription of OSBP2 and FABP3, fatty acid-binding protein 3, was increased upon OSW-1 treatment (Fig. 1*A* and Table 1). To investigate whether PI4P levels in the *trans* Golgi regions are responsible for the transcriptional induction of OSBP2 and FABP3, we examined their transcription in PITPNB KO and PI4KB KO cells by qRT-PCR experiments (Fig. 5). As expected, the transcriptional induction of OSBP2 and FABP3 was diminished in PITPNB KO (Fig. 5, *A* and *B*) or PI4KB KO cells (Fig. 5, *C* and *D*). These data suggest that transcriptional induction of OSBP2 and FABP3 by the PI4P pathway is triggered depending on PI4P levels in the *trans* Golgi regions as observed in cell death induced by OSW-1.

### Either PITPNB or PI4KB is indispensable for Golgi fragmentation induced by OSW-1

**Figure 6:**
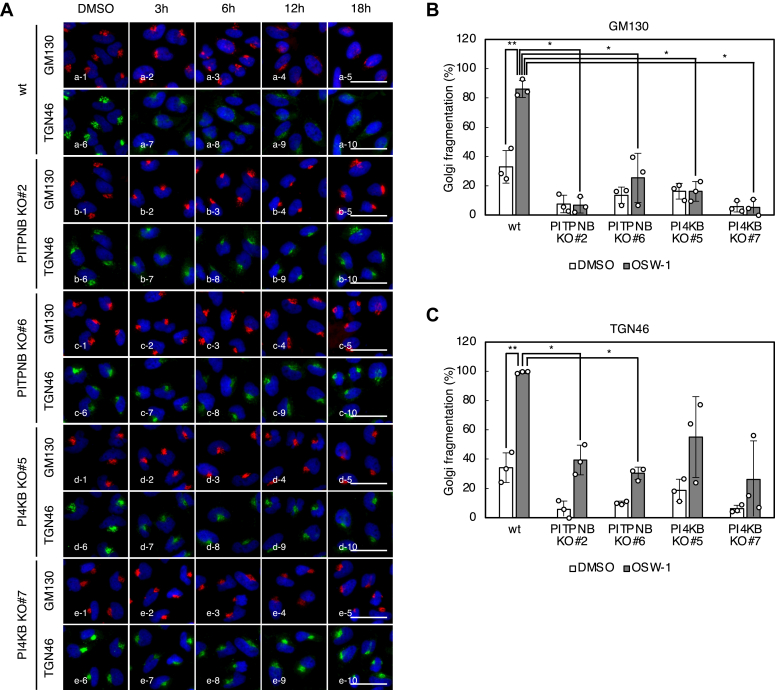
**Golgi fragmentation by OSW-1 in PITPNB KO or PI4KB KO cells**. *A*, immunofluorescence microscopic analysis of wt HeLa cells, PITPNB KO clones (#2 and #6), and PI4KB KO clones (#5 and #7) treated with or without 5 nM OSW-1 for the indicated time periods. Cells were stained with murine anti-GM130 mAb (*red*), sheep anti-TGN46 pAb (*green*), and DAPI (*blue*). The scale bars represent 50 μm. *B* and *C*, quantification of Golgi fragmentation in wt HeLa cells, PITPNB KO clones (#2 and #6), and PI4KB KO clones (#5 and #7) treated with or without 5 nM OSW-1 for 18 h. The percentage of cells with fragmented Golgi was calculated using images immunostained with anti-GM130 mAb or anti-TGN46 pAb. ∗*p* < 0.05, ∗∗*p* < 0.01 (the Bonferroni-corrected *t* test; mean ± S.D.; n = 3). DAPI, 4′,6-diamidino-2-phenylindole; mAb, monoclonal antibody; PITP, (PI)-transfer protein; PI4K, PI 4-kinase; pAb, polyclonal antibody; TGN, *trans* Golgi network.

Previously, it was reported that OSW-1 treatment induces Golgi fragmentation before cell death (19). To further investigate whether PI4P levels in the *trans* Golgi regions are responsible for Golgi fragmentation caused by OSW-1, we performed immunostaining using antibodies against GM130 and TGN46, which are a *cis*-Golgi marker and a TGN marker, respectively (Fig. 6). In wt HeLa cells, GM130 and TGN46 were accumulated in the perinuclear region under the normal condition (Fig. 6*A*, panels a-1 and a-6), whereas they began to disperse about 6 h later upon the treatment of OSW-1 (panels a-3 ∼ 5 and a-8 ∼ 10). Unexpectedly, we did not observe the remarkable dispersion of GM130 and TGN46 even 18 h after OSW-1 treatment in PITPNB or PI4KB KO cell lines (panels b-3 ∼ 5, b-8 ∼ 10, c-3 ∼ 5, c-8 ∼ 10, d-3 ∼ 5, d-8 ∼ 10, e−3 ∼ 5, and e−8 ∼ 10). Statistically significant decreases in the percentage of cells showing *cis*-Golgi fragmentation by OSW-1 were observed in PITPNB or PI4KB KO cells compared to wt cells (ratio of *cis*-Golgi-fragmented cells: wt, 86 ± 3.3%; PITPNB KO#2, 6.8 ± 3.2%; PITPNB KO#6, 25 ± 9.7%; PI4KB KO#5, 16 ± 3.9%; and PI4KB KO#7, 5.2 ± 3.2%) (Fig. 6*B*). Similarly, decreases in the percentage of cells showing TGN fragmentation by OSW-1 were observed in PITPNB or PI4KB KO cells (ratio of TGN-fragmented cells: wt, 99 ± 0.56%; PITPNB KO#2, 39 ± 5.9%; PITPNB KO#6, 30 ± 2.5%; PI4KB KO#5, 55 ± 16%; and PI4KB KO#7, 26 ± 15%) (Fig. 6*C*). Furthermore, we compared differences in microstructural changes of the Golgi induced by OSW-1 between wt HeLa cells and PITPNB KO cells using 3D reconstruction analysis. In wt HeLa cells, the Golgi was fragmented by OSW-1 and multiple vesicles derived from the Golgi were detected throughout the cytoplasm (Movies S1 and S2). On the other hand, in PITPNB KO cells, the Golgi had been already partially fragmented even without OSW-1 (Movie S3) and OSW-1 treatment did not significantly alter the microstructure of the Golgi and no vesiculation was observed (Movie S4). These data indicate that both PITPNB and PI4KB are indispensable for Golgi fragmentation induced by OSW-1.

### GOLPH3 is not involved in Golgi fragmentation induced by OSW-1

**Figure 7:**
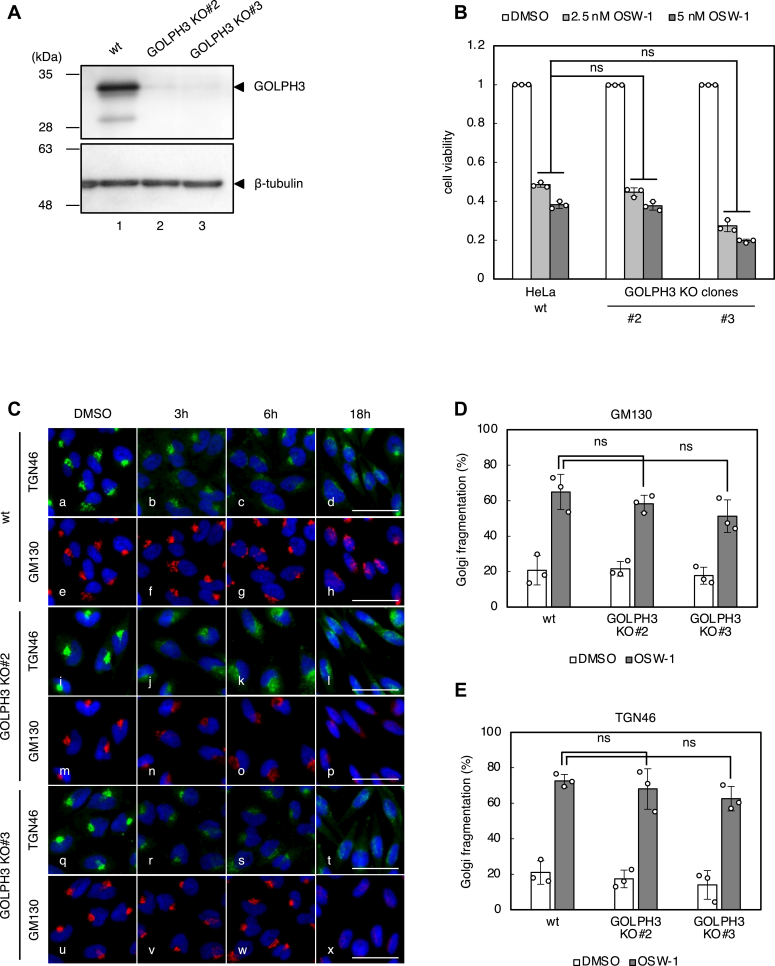
**Cell death and Golgi fragmentation by OSW-1 in GOLPH3 KO cells**. *A*, immunoblot analysis of GOLPH3 protein in wt HeLa cells and two individual clones (#2 and #3) of GOLPH3 KO cells. β-tubulin was used as a loading control. *B*, cell viability assay. Wt HeLa cells and GOLPH3 KO clones (#2 and #3) were treated with or without the indicated concentrations of OSW-1 for 48 h, and subjected to cell viability assay. ns, not significant (the Bonferroni-corrected *t* test; mean ± S.D.; n = 3). *C*, immunofluorescence microscopic analysis of wt HeLa cells and GOLPH3 KO clones (#2 and #3) treated with or without 5 nM OSW-1 for the indicated time periods. Cells were stained with murine anti-GM130 mAb (*red*), sheep anti-TGN46 pAb (*green*), and DAPI (*blue*). The scale bars represent 50 μm. *D* and *E*, quantification of Golgi fragmentation in wt HeLa cells and GOLPH3 KO clones (#2 and #3) treated with or without 5 nM OSW-1 for 18 h. The percentage of cells with fragmented Golgi was calculated using images immunostained with anti-GM130 mAb or anti-TGN46 pAb. ns, not significant (the Bonferroni-corrected *t* test; mean ± S.D.; n = 3). DAPI, 4′,6-diamidino-2-phenylindole; mAb, monoclonal antibody; pAb, polyclonal antibody; TGN, *trans* Golgi network.

Farber-Katz et al., previously reported that DNA damage induces Golgi dispersion throughout the cytoplasm, which is regulated by GOLPH3/MYO18A/F-actin and the DNA damage protein kinase, DNA-PK (34). GOLPH3, which is localized at the TGN through PI4P-binding, is phosphorylated by DNA-PK upon DNA damage. This phosphorylation increases the interaction between GOLPH3 and MYO18A and applies a tensile force to induce the Golgi dispersion. To investigate whether GOLPH3 regulates Golgi fragmentation and cell death caused by OSW-1, we first established two stable cell lines of GOLPH3 KO cells (GOLPH3 KO#2 and #3). We confirmed that the expression of GOLPH3 protein was completely lost using immunoblotting analysis (Fig. 7*A*, lanes 2 and 3). OSW-1 induced cell death in GOLPH3 KO#2 and KO#3 cells to the same extent as wt cells (Fig. 7*B*). Moreover, depletion of GOLPH3 did not suppress Golgi fragmentation induced by OSW-1 (Fig. 7*C*) and statistically significant decreases in the percentage of cells showing *cis*-Golgi fragmentation and TGN fragmentation by OSW-1 were not detected in GOLPH3 KO cells compared to wt cells (Fig. 7, *D* and *E*). Golgi dispersion is required for cell survival after DNA damage triggered by anticancer drugs such as camptothecin and doxorubicin. In contrast, OSW-1-induced Golgi fragmentation is likely to be a key step leading to cell death because Golgi fragmentation after OSW-1 treatment did not occur in addition to cell death in PITPNB- or PI4KB-depleted cells. Thus, the mechanism of Golgi fragmentation by OSW-1 seems to be different from that of Golgi fragmentation by DNA damage.

### OSW-1 abolishes glycosylation in the Golgi depending on PI4P levels in the *trans* Golgi regions

**Figure 8:**
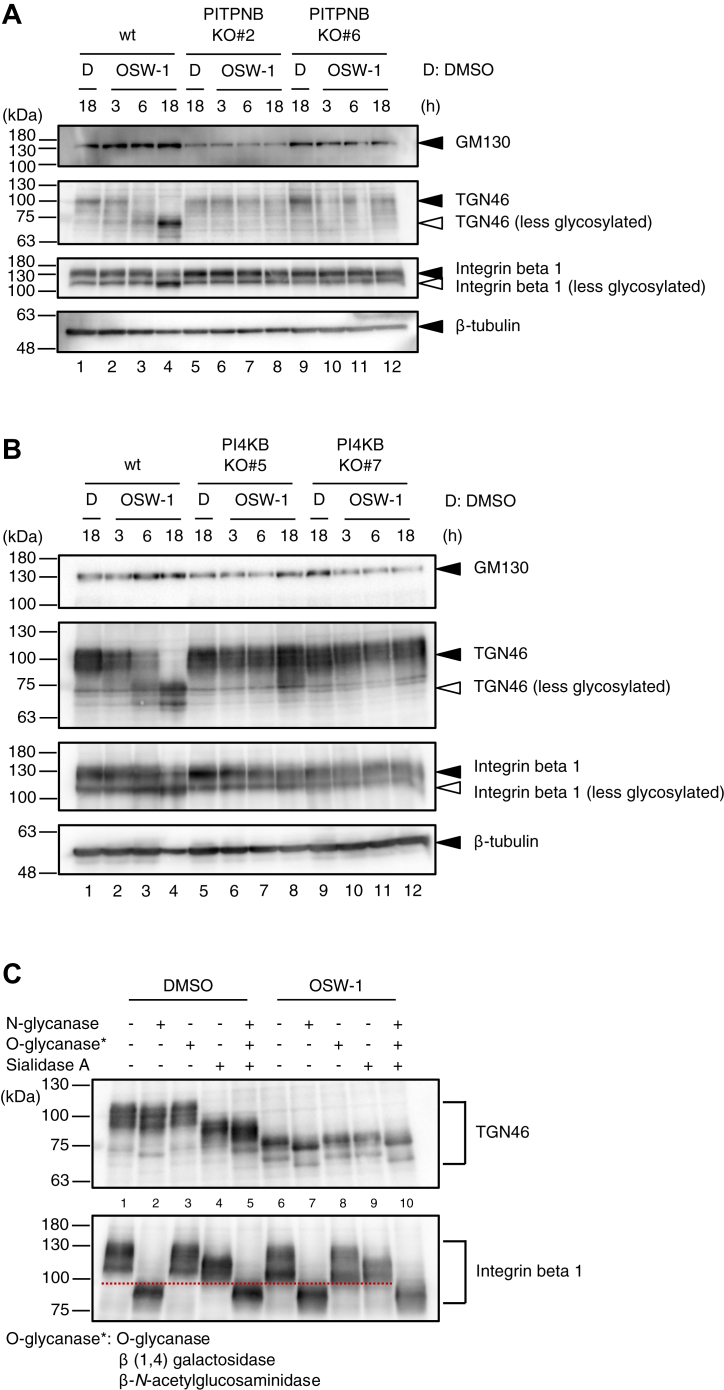
**Glycosylation in the Golgi of PITPNB KO and PI4KB KO cells**. *A* and *B*, immunoblot analysis of GM130, TGN46, and integrin β1 proteins. Whole cell lysates prepared from wt HeLa cells and PITPNB KO cells (*A*) or PI4KB KO cells (*B*), treated with or without 5 nM OSW-1 for 18 h, were subjected to immunoblot analysis. β-tubulin was used as a loading control. *C*, deglycosylation assay of TGN46 and integrin β1 proteins in wt HeLa cells treated with or without 5 nM OSW-1 for the indicated time periods. Deglycosylation enzymes used in this assay were as follows: *N*-glycanase, *O*-glycanase mix (*O*-glycanase, β (1–4) galactosidase and β-*N*-acetylglucosaminidase), and sialidase A. PITP, (PI)-transfer protein; PI4K, PI 4-kinase; TGN, *trans* Golgi network.

To investigate the events in the Golgi fragmented by OSW-1 treatment, immunoblotting analysis was performed using anti-GM130 and anti-TGN46 antibodies. The molecular weight of GM130 did not change after OSW-1 treatment in wt HeLa cells (Fig. 8, *A* and *B*, upper panel, lanes 1–4), whereas that of TGN46 markedly shifted from 100 kDa to 75 kDa (second panel, lanes 1–4).

To investigate whether the molecular weight shift of TGN46 caused by OSW-1 treatment was due to insufficient glycosylation, we examined the glycosylation state of TGN46, which has been reported to be a glycoprotein with *N*- and *O*-linked glycans (35, 36). Whole cell lysates of wt HeLa cells were treated with *N*-glycanase to remove *N*-glycan, *O*-glycanase, β (1, 4) galactosidase, and β-*N*-acetylglucosaminidase to degrade *O*-glycan, and sialidase A to remove sialic acid, and subjected to immunoblotting (Fig. 8*C*). The treatment of these deglycosylation enzymes reduced the molecular weight of TGN46 (lanes 1 and 5), indicating that TGN46 is highly glycosylated. Even after OSW-1 treatment, the molecular weight of TGN46 was further reduced by *N*-glycanase treatment (lanes 6 and 7), indicating that the *N*-glycosylation, which occurs in the ER, was not affected by OSW-1. In contrast, the molecular weight of TGN46 was not changed by *O*-glycanase, β (1, 4) galactosidase, β-*N*-acetylglucosaminidase, and sialidase A treatments (lanes 6, 8, and 9). This indicates that the sialidation and *O*-linked glycosylation, which occurs in the Golgi, was inhibited by OSW-1. We also investigated the glycosylation state of another protein, integrin β1. Integrin β1 is a highly glycosylated protein containing multiple *N*-linked glycans. The upper bands of integrin β1 were decreased, and the lower bands were increased after OSW-1 treatment in wt HeLa cells (third panel, lanes 1–4). *N*-glycanase treatment significantly reduced the molecular weight of integrin β1 in the presence or absence of OSW-1 (lanes 1, 2, 6, and 7). On the other hand, *O*-glycanase, β (1, 4) galactosidase, and β-*N*-acetylglucosaminidase treatments did not change the molecular weight of integrin β1 (lanes 1, 3, 6, and 8). The bands of integrin β1 shifted downward up to the red dashed line after treatment with sialidase A in the absence of OSW-1 (lanes 1 and 4), however, in the presence of OSW-1, the band of integrin β1 untreated with any glycosidases (lane 6) had already shifted downward up to the red dashed line without sialidase A, indicating that OSW-1 inhibits glycosylation in the Golgi such as sialidation but not *N*-glycosylation in the ER. Interestingly, the molecular weight of TGN46 and integrin β1 returned to normal in PITPNB KO and PI4KB KO cells treated with OSW-1 (Fig. 8, *A* and *B*, lanes 5–12), suggesting that OSW-1 represses Golgi functions such as glycosylation depending on PI4P levels in the *trans* Golgi regions.

### Analysis of PI4P levels in the *trans* Golgi regions under the presence of OSW-1 in PITPNB KO cells and PI4KB KO cells

**Figure 9:**
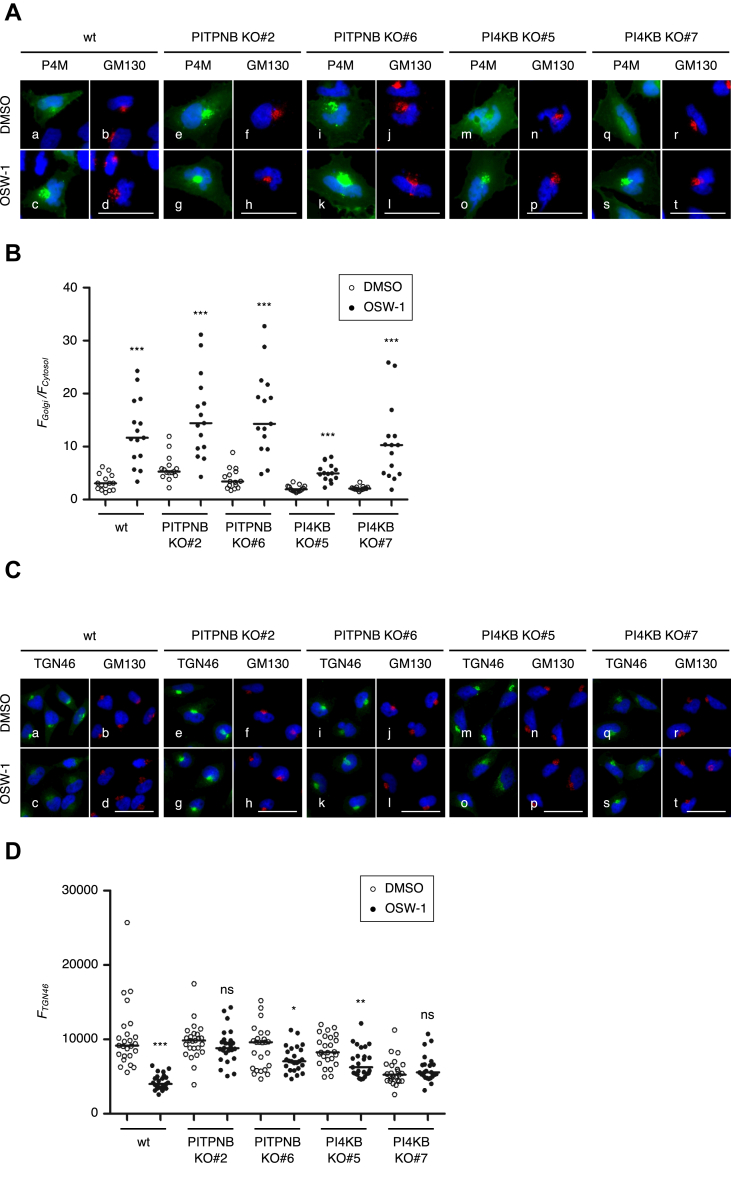
**PI4P levels in the *trans* Golgi regions of PITPNB KO and PI4KB KO cells**. *A*, microscopic observation of GFP-P4M (P4M) in the *trans* Golgi regions. Wt HeLa cells, PITPNB KO clones (#2 and #6), and PI4KB KO clones (#5 and #7) were transfected with an expression plasmid of GFP-P4M and treated with or without 20 nM OSW-1 for 1 h. Cells were stained with murine anti-GM130 mAb (*red*) and with DAPI (*blue*). The scale bars represent 50 μm. *B*, quantification of the fluorescent intensity of GFP-P4M-SidM in the *trans* Golgi regions of wt HeLa cells, PITPNB KO clones (#2 and #6) and PI4KB KO clones (#5 and #7) treated with or without 20 nM OSW-1 for 1 h. The *dots* represent the ratios of the fluorescent intensity of GFP-P4M-SidM in the *trans* Golgi regions to those in the cytosol. *Central lines* represent the median values (n = 15 cells). ∗∗∗*p* < 0.001 (Student’s *t* test). *C*, immunofluorescence microscopic analysis of wt HeLa cells, PITPNB KO clones (#2 and #6), and PI4KB KO clones (#5 and #7) treated with or without 20 nM OSW-1 for 1 h. Cells were stained with murine anti-GM130 mAb (*red*), sheep anti-TGN46 pAb (*green*), and DAPI (*blue*). The scale bars represent 50 μm. *D*, quantification of the fluorescent intensity of TGN46 in wt HeLa cells, PITPNB KO clones (#2 and #6), and PI4KB KO clones (#5 and #7) treated with or without 20 nM OSW-1 for 1 h. The *dots* represent the fluorescent intensity of TGN46, which was calculated using images immunostained with anti-TGN46 pAb. *Central lines* represent the median values (n = 25 cells). ∗*p* < 0.05, ∗∗*p* < 0.01, ∗∗∗*p* < 0.001 (Student’s *t* test). ns, not significant. DAPI, 4′,6-diamidino-2-phenylindole; mAb, monoclonal antibody; PI4P, phosphatidylinositol-4-phosphate; PITP, (PI)-transfer protein; PI4K, PI 4-kinase; pAb, polyclonal antibody; TGN, *trans* Golgi network.

Since OSW-1 inhibits the exchange transport of cholesterol and PI4P between the ER and the Golgi by OSBP, PI4P levels in the TGN are increased upon OSW-1 treatment (18). We hypothesized that an increase in PI4P levels in the *trans* Golgi regions upon OSW-1 treatment is responsible for Golgi fragmentation, inhibition of glycosylation, transcriptional induction, and cell death. Thus, we evaluated PI4P levels in the *trans* Golgi regions by expressing GFP-P4M-SidM as a reporter of PI4P, which is the GFP-fused SidM (P4M) domain derived from the bacterial pathogen *Legionella pneumophila* (37) (Fig. 9). PI4P levels in the *trans* Golgi regions of wt HeLa cells were markedly elevated by OSW-1 treatment for 1 h (Fig. 9, *A* panels a and c, and *B*). Unexpectedly, PI4P levels in PITPNB KO and PI4KB KO cells were also significantly increased as observed in wt cells (Fig. 9, *A* panels e, g, i, k, m, o, q, and s, and *B*), which seems inconsistent with our hypothesis. Our explanation for this discrepancy is that PI4P levels of wt cells can be underestimated in microscopic analysis due to the fragmentation of the *trans* Golgi regions. TGN46 levels of wt cells appear to be reduced 1 h after OSW-1 treatment under microscopic observation (Fig. 9, *C* panels a and c, and *D*), but those are actually not changed when examined by immunoblotting (Fig. 8, *A* and *B*). In contrast, TGN46 levels of PITPNB KO and PI4KB KO cells were not markedly altered by OSW-1 compared to wt (Fig. 9, *C* panels e, g, i, k, m, o, q, and s, and *D*). We speculated that signals of PI4P and TGN46 become scattered and blow the detection limit in fragmented Golgi, resulting only in the appearance of reduced signals in wt cells, though the actual PI4P levels in wt cells are much higher enough to kill cells than those in PITPNB KO cells and PI4KB KO cells.

## Discussion

**Figure 10:**
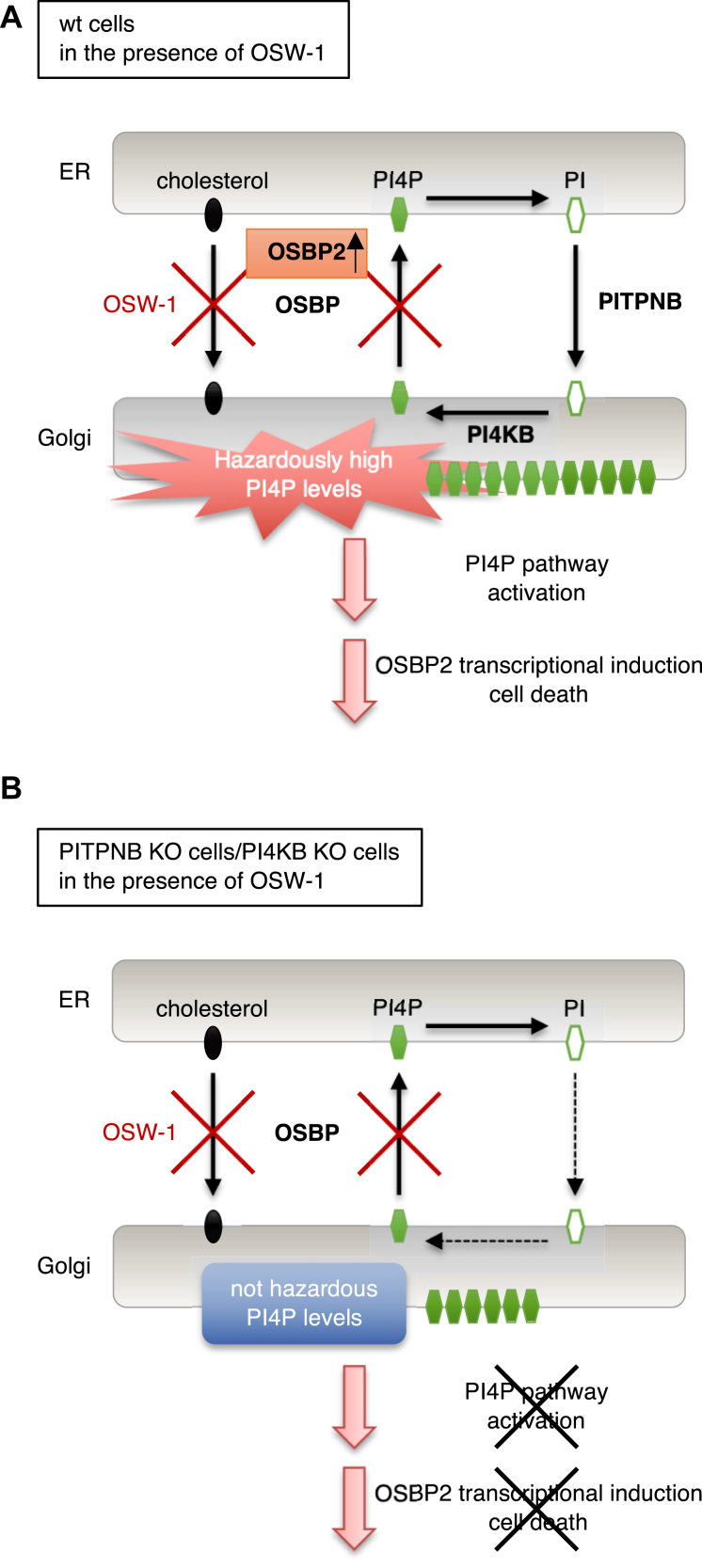
**Our working hypothesis for the activation of the PI4P pathway of the Golgi stress response**. *A*, In wt cells, treatment of OSW-1 inhibits OSBP, leading to the accumulation of PI4P in the *trans* Golgi regions, which causes Golgi fragmentation, OSBP2 transcriptional induction through the activation of the PI4P pathway and cell death. *B*, in contrast, PITPNB or PI4KB KO cells are presumed to exhibit lower levels of PI4P upon treatment of OSW-1 since the PITPNB and PI4KB genes are involved in PI4P synthesis. Therefore, in PITPNB KO or PI4KB KO cells, OSW-1 treatment seems not to induce overaccumulation of PI4P at concentrations high enough to activate the PI4P pathway. As a result, cell death is suppressed in these KO cells. OSBP, oxysterol-binding protein; PI4P, phosphatidylinositol-4-phosphate; PI4K, PI 4-kinase; PITP, (PI)-transfer protein.

In this study, we revealed that OSW-1 treatment evokes fragmentation of the Golgi, inhibition of glycosylation in the Golgi, transcriptional induction of OSBP2, and cell death. Disruption of genes involved in PI4P synthesis in the *trans* Golgi regions including PITPNB and PI4KB canceled these effects of OSW-1. Based on these observations, we propose that OSW-1 induces transcription and cell death by activating a novel pathway of the Golgi stress response, the PI4P pathway, through dysregulation of the PI4P metabolism in the *trans* Golgi regions (Fig. 10).

We performed RNA-seq (Fig. 1*A*, Tables 1, and 2) and qRT-PCR analyses (Fig. 1, *B* and *C*) of HeLa cells treated with or without OSW-1, and found that transcription of the OSBP2 and FABP3 genes is increased upon OSW-1 treatment (Fig. 5), while we did not detect an increase of OSBP mRNA expression (Fig. 1*B*). The PH and ORD domains of OSBP2 bind to PI4P and cholesterol, respectively (38), OSBP2 can extract and transfer cholesterol between liposomes in invitro systems (38), and OSBP2 is localized to the Golgi by forming a heterodimer with OSBP (21), suggesting that OSBP2 transports cholesterol and PI4P between the ER and the Golgi like OSBP, though it should be determined by direct experiments. Since OSW-1 binds to the ORD of OSBP with higher affinity than that of OSBP2 (39), OSBP2 may not be completely inhibited by OSW-1. Thus, it is possible that OSBP is a constitutively expressed type, while OSBP2 is an inducible one to compensate for the cholesterol/PI4P transfer activity between the ER and the Golgi. FABP3 belongs to the FABP family, which consists of nine members that are expressed in a tissue-specific manner, and is highly expressed in the heart and skeletal muscles (40). FABPs bind to long-chain fatty acids and are considered to play a role in the transport of lipids to intracellular compartments such as lipid droplets and organelles for storage, metabolism and signaling, and extracellular regions for signaling (40). FABP3 is upregulated especially in aged skeletal muscles, reduces membrane fluidity by increasing lipid saturation and induces ER stress (41). The upregulation of FABP3 expression observed here may reflect a disturbance of lipid composition in the Golgi membrane. The mammalian Golgi stress response consists of several response pathways including the TFE3 pathway, the proteoglycan pathway, the mucin pathway, the CREB3 pathway, and the HSP47 pathway (7). Since OSBP2 and FABP3 are not included in the list of target genes of these well-known pathways, the transcriptional induction of the OSBP2 and FABP3 is thought to be regulated by a novel response pathway of the Golgi stress response, that is, the PI4P pathway, which is activated by metabolic dysregulation of PI4P in the *trans* Golgi regions.

PITPNB, C10orf76, CDIPT, and PI4KB were identified as candidates essential for cell death induced by OSW-1 (Fig. 2*A*). In addition, one sgRNA targeting PI4K2A was enriched in GeCKO screening using OSW-1-induced cell death (Fig. 2*C*). All of these proteins are involved in the synthesis of PI4P in the *trans* Golgi regions (Fig. 2*B*). PITPNB encodes PITPβ, which belongs to the PITP protein family with one common PITP domain and has exchange activity of phosphatidylinositol (PI)/phosphatidylcholine or PI/phosphatidic acid between different organelles (26). The PITP family includes five proteins: PITPα, PITPβ, PITPNM1, PITPNM2, and PITPNC1. Among them, PITPα and PITPβ share the most homologous common structure and are ubiquitously expressed in mammals. Xie *et al.* found that both PITPα and PITPβ regulate the production of the PI4P pool at the Golgi, which is essential for the normal development of the neocortex in mammalian embryos (42). However, our GeCKO screen did not identify any genes encoding members of the PITP protein family other than PITPβ (Fig. 2*C*). It may be because PITPβ shuttles between the ER and the Golgi, while PITPα resides in the cytosol (43). Although PITPNC1 has also been shown to maintain a pool of PI4P in the Golgi, there are no reports comparing the contribution of these proteins to the production of PI4P. PITPβ, together with PI4P metabolic regulators such as OSBP, PI4KB, and SAC1, is localized to the MCS between the ER and the Golgi (Fig. 2*B*) and is presumed to actively contribute to the regulation of PI4P levels in the Golgi of mammalian cells.

PI4KB and PI4K2A are PI4Ks that generate a pool of PI4P in the Golgi (29, 44). CDIPT protein catalyzes the *de novo* synthesis of PI (27, 28). C10orf76 forms a direct complex with PI4KB and contributes PI4KB activation *via* the recruitment of ARF1-GTP to the Golgi. KO of C10orf76 reduces PI4P levels in the Golgi in HAP1 cells (32, 45) and in HeLa cells (33). From these, it is inferred that inhibition of PI4P transport from the *trans* Golgi regions to the ER by OSW-1 leads to excessive accumulation of PI4P in the *trans* Golgi regions and drastic change in the lipid composition of the Golgi membrane, resulting in the collapse of the Golgi and activation of the PI4P pathway of the Golgi stress response in wt cells. The PI4P pathway may induce transcription of OSBP2 to alleviate PI4P accumulation, and prolonged Golgi stress may ultimately cause cell death (Fig. 10*A*). In fact, it is reported that lysosomal PI4P accumulation caused by depletion of OSBP resulted in cell death during lysosomal damage repair although the reason has been unclear yet (46). On the other hand, PI4P levels in PI4KB, PI4K2A, PITPNB, CDIPT, or C10orf76 KO cells treated with OSW-1 should be lower than those in wt cells and could not activate the PI4P pathway of the Golgi stress response (Fig. 10*B*). Recently, two kinds of inhibitors against OSBP were identified (47, 48). One is oxybipin, which is a sterol-based chemical chimera and leads to the blockage of retrograde trafficking and the degradation of several Golgi proteins (47). The other is orpinolide, which is a synthetic withanolide analog with pronounced antileukemic properties and disrupts Golgi homeostasis (48). The effects of these inhibitors on the Golgi function and cell viability were attenuated in the presence of selective inhibitors of PI4KB such as BF738735 and MI14. These results confirm the credibility of our model that a rapid increase in PI4P levels in the *trans* Golgi regions may cause Golgi stress, leading to cell death. Although it has been already reported that a decrease in PI4P levels weakens Golgi function (49), this is the first report that excessive accumulation of PI4P in the *trans* Golgi regions represses Golgi function. The molecular mechanism that senses PI4P accumulation and activates the PI4P pathway of the Golgi stress response is still unknown and clarification of this sensing mechanism is an important issue for the future research.

Although OSW-1 exerts potent and selective anticancer effects, the question of how inhibition of OSBP by OSW-1 leads to cell death specific to cancer cells has remained unresolved for a long time. Increased expression of PI4P-related factors localized to the Golgi, such as PI4KB, SAC1, GOLPH3, and PITPNC1, is one of the common features of several tumors (50). For instance, in some tumors, PI4KB is overexpressed due to the multiplication of the PI4KB gene, resulting in the overproduction of PI4P in the *trans* Golgi regions. If PI4P is increased in the *trans* Golgi regions, PI4P-binding proteins like GOLPH3 and PITPNC1 are over-recruited, leading to malignant secretion that can develop angiogenesis, tumor invasion, and metastasis. A strong correlation between high levels of PI4P at the Golgi and the invasive phenotype of breast cancer cell lines was also reported (51, 52). According to these reports, decreased Golgi PI4P levels by knockdown of PI4KB in highly invasive cells such as MDA-MB-231 decreased the invasive phenotype. In contrast, increased Golgi PI4P levels by knockdown of SAC1 in weakly invasive cells such as MCF7 showed the reverse effects. Thus, we speculate that the anticancer effect of OSW-1 is derived from the increase of PI4P levels in the Golgi of cancer cells. This is consistent with our finding that the depletion of factors related to PI4P synthesis in the Golgi, such as PITPNB and PI4KB, leads to resistance to OSW-1. In addition to conventional anticancer drugs, the development of cancer medicines with various molecular targets is in progress worldwide. Since cancer cells continuously mutate, change their properties, and eventually acquire drug resistance, further exploration of a wide range of molecular targets to kill cancer cells is essential to solving the problem of drug resistance. In this study, we clearly revealed that OSW-1 is a unique anticancer reagent that targets the Golgi function. Our discovery will not only elucidate the role of the Golgi in the malignant transformation of cancer but it will also lead to the development of Golgi-based cancer therapies in the future.

## Experimental procedures

### Antibodies and reagents

The following antibodies were purchased: rabbit anti-PITPNB polyclonal antibody (pAb) and sheep anti-TGN46 pAb from Sigma-Aldrich; rabbit anti-β-tubulin pAb from cell signaling; rabbit anti-PI4KB pAb, rabbit anti-GOLPH3 pAb, and rabbit anti-integrin β1 pAb from Abcam; mouse anti-GM130 monoclonal antibody (mAb) from BD Biosciences. OSW-1 was synthesized as described previously (53). Thapsigargin and monensin were obtained from Calbiochem. PIK-93 was purchased from ChemScene.

### Plasmid construction

Human PITPNB cDNA (OHu06208C) was purchased from GenScript. GFP-P4M-SidM was purchased from Addgene (plasmid #51469). To construct plasmids for KO of PITPNB, PI4KB, and GOLPH3 genes, each oligo DNA fragment for expression of corresponding sgRNA was inserted into the BbsI site of pSpCas9 (BB)-2A-Puro (PX459) V2.0, which was purchased from Addgene (plasmid #62988).

### Cell culture and transfection

HeLa cells were cultured in Dulbecco’s modified Eagle’s medium (glucose at 4.5 g/liter) (Nacalai Tesque) supplemented with 10% fetal bovine serum (FBS) and Penicillin-Streptomycin Solution (Wako) at 37 °C under humidified air containing 5% CO_2_. Cells were seeded and cultured for 1 day, and then they were transfected with siRNA duplexes targeting OSBP (siOSBP-a, 5′-GCAAUGACUUGAUAGUAAtt-3′; siOSBP-b, 5′-GGAGACAAGUGUAAUCUUAtt-3′; and siOSBP-c, 5′-GGAAUACCAUGAGCUGUUAtt-3′; purchased from Ambion) using RNAiMAX (Invitrogen) or the expression plasmid DNAs using FuGENE 6 (Promega).

### RNA sample preparation, RNA-seq, and quantitative RT-PCR

Cells were harvested 18 h after the treatment with 5 nM OSW-1, and then total RNA was prepared with Sepasol Super G (Nacalai Tesque). RNA-seq was performed with the Ion Proton System for Next Generation Sequencing with PI chips (Thermo Fisher Scientific). RT-PCR and quantitative PCR were performed using a PrimeScript RT reagent kit with gDNA Eraser, SYBR Premix Ex Taq II (TliRNase H Plus) (TaKaRa) and an ABI7500 qPCR instrument (Life Technologies). Primer pairs used for qRT-PCR were as follows: *OSBP* (GCAGGGTCATGCTGTGGAA and CATCAGTCTCTGGTCAGGTCGTAA), *OSBP2* (GTACCAGACCCTGTCAGCCAAG and GTCAGGGCCAGCTCTGAGAA), *FABP3* (TGCGGGAGCTAATTGATGGAA and TTCTCATAAGTGCGAGTGCAAACTG), and *GAPDH* (TGCACCACCAACTGCTTAGC and GGCATGGACTGTGGTCATGAG).

### GeCKO screening

Two sgRNA-expressing HeLa cell libraries (A-1 and A-2) using the GeCKO v2.0 library (Libraries A) were previously prepared (54). sgRNA-expressing cells were treated with 5 nM OSW-1 for 48 h. Then, cells were washed with Dulbecco's modified Eagle's medium containing 10% FBS and cultured in conventional conditions for 11 days. Surviving cells were replated and treated with 5 nM OSW-1 again for 48 h. Five days after treatment, cells were trypsinized and frozen as cell pellets. For untreated controls, sgRNA-expressing cells were cultured for the same period as OSW-1-treated cells with several passages.

### Isolation of genomic DNA and genome-integrated sgRNA sequencing

Analysis of genome-integrated sgRNAs was performed as described previously (54). Genomic DNA from frozen cells was purified using the conventional phenol-chloroform method. The genome-integrated sgRNA sequences were amplified using KOD-Plus-Neo (Toyobo), and 100 μg genomic DNA from untreated cells or OSW-1-treated cells as a PCR template. Primer pairs used for the PCR were as follows: TCTTGTGGAAAGGACGAAACACCG and GCCACTTTTTCAAGTTGATAACGGACTAG. The amplicons were resolved by agarose electrophoresis and extracted from the gel using NucleoSpin Gel and PCR Clean-up XS (Macherey-Nagel). The extracted DNA was then processed into libraries for sequencing using Ion Plus Fragment Library Kit and Ion Xpress Barcode Adapters 1 to 16 (Thermo Fisher Scientific). Sequencing was performed using an Ion Proton System (Thermo Fisher Scientific), and then data processing and analysis were carried out using an SgRNA Screening v.1.1 custom plugin (Thermo Fisher Scientific).

### Generation of KO cell lines

Generation of PITPNB, PI4KB, and GOLPH3 KO HeLa cells was carried out using the CRISPR/Cas9 system as described previously (55). Briefly, HeLa cells were transfected with the plasmid (pSpCas9 (BB)-2A-Puro (PX459) V2.0/PITPNB sgRNA or pSpCas9 (BB)-2A-Puro (PX459) V2.0/PI4KB sgRNA) using FuGENE 6. After 24 h, cells were selected with 1 μg/ml puromycin for 3 days to remove untransfected cells. Subsequently, cells were plated on 96-well plates in media without puromycin, and single colonies were selected.

### Indel analysis

Isolation of genomic DNA for PCR was performed as described previously (56). PCR was performed with KOD-Plus-Neo (Toyobo), and blunt-end PCR products were treated with 10 × A-attachment mix (Toyobo) to acquire overhanging dA at the 3′-ends. Products with 3′-dA overhangs were cloned into T-Vector pMD19 (Simple) (Takara Bio Inc) to use as a template for sequencing.

### Cell viability assay

Cell viability assay was performed using a Cell Counting Kit-8 (DOJINDO) according to the manufacturer’s instructions. Briefly, HeLa cells were treated with 5 nM OSW-1, 1 μM thapsigargin, or 10 μM monensin for 48 h and incubated with CCK-8 solution in the culture medium for 4 h at 37 °C in a CO_2_ incubator. The absorbance at 415 nm was measured using a microplate reader, Sunrise (TECAN).

### Western blot analysis

Western blot analysis was performed as described previously (57). Whole cell lysates were resolved by SDS-PAGE, transferred to polyvinylidene difluoride membranes (Immobilon-P, Merck Millipore), and probed with specific antibodies. Chemiluminescent signals were detected by a WSE-6300 LuminoGraph III (ATTO) using EZ-ECL chemiluminescence detection kit for horseradish peroxidase (Sartorius).

### Immunofluorescence microscopic analysis

HeLa cells were seeded onto coverslips in 24-well plates, washed with PBS, fixed in 4% (w/v) paraformaldehyde in PBS at room temperature for 15 min, and then permeabilized in 0.2% Triton X-100 in PBS at room temperature for 10 min. After blocking with 10% FBS in PBS at room temperature for 1 h, the cells on the coverslips were incubated with primary antibodies at 4 °C overnight and then with secondary antibodies (Alexa Fluor 488-labeled donkey anti-sheep immunoglobulin G (IgG) used for TGN46 staining and Alexa Fluor 555-labeled donkey anti-mouse IgG used for GM130 staining) at room temperature for 1 h. Finally, they were mounted with SlowFade Gold antifade reagent (Invitrogen) and their images were acquired using an Eclipse Ni microscope (Nikon) and ORCA-ER digital CCD camera (Hamamatsu Photonics). Immunostaining of HeLa cells expressing GFP-P4M-SidM was performed with reference to immunostaining of Golgi-localizing PI4P with anti-PI4P antibody (58). They were fixed in 2% (w/v) paraformaldehyde in PBS at room temperature for 15 min, and then permeabilized in 20 μM digitonin in PBS at room temperature for 5 min. After blocking with 5% normal goat serum in PBS at room temperature for 1 h, the cells on the coverslips were incubated with mouse anti-GM130 mAb at 4 °C overnight and then with Alexa Fluor 555-labeled donkey anti-mouse IgG at room temperature for 1 h.

Quantification of Golgi fragmentation was performed as described previously (59). In brief, the random fields of GM130 or TGN46 images in immunostaining were analyzed using ImageJ Fiji (https://imagej.net/software/fiji/). For each field, GM130 or TGN46 signals were automatically selected by thresholding and normal/unfragmented Golgi (Size: 500-Infinity, Circularity: 0.20–1.00) was counted using the Analyze particles tool. Quantification of the fluorescent intensity of GFP-P4M and TGN46 was also performed using ImageJ Fiji, and the Golgi regions were selected based on the GM130 signals.

### Electron microscopic analysis

HeLa cells were treated with or without 5 nM OSW-1 for 18 h and washed with PBS, fixed in 0.5% (w/v) paraformaldehyde and 0.5% glutaraldehyde in PBS at room temperature for 20 min. Cells were embedded in agarose as previously described (60). Specimens for the osmium maceration method were prepared as previously described (61). Finally, cells were observed in field emission scanning electron microscopes (S4100 and Regulus, Hitachi-High-Tech). Samples for the serial section SEM were prepared according to our article (61). The sections were observed in an ultra-high-resolution SEM (SU-70, Hitachi High-Tech) to acquire sequential images of target cells. After aligning the serial images using computer software (Amira, Thermo Fisher Scientific), target structures such as the Golgi apparatus, nuclei, and cytoplasm were segmented and reconstructed in three dimensions. Immunoelectron microscopy analysis was performed as described previously (62).

### Deglycosylation assay

Deglycosylation assay was performed as described previously (35). In brief, HeLa cells were treated with or without 5 nM OSW-1 for 18 h and lysed in radio-immunoprecipitation assay buffer supplemented with protease inhibitor cocktail, cOmplete, EDTA-free (Roche), and proteins were quantified using BCA Protein Assay Kit (Thermo Fisher Scientific). The reaction of deglycosylation was performed with the Agilent Enzymatic Deglycosylation Kit for *N*-Linked and Simple *O*-Linked Glycans (GK80110) and Agilent Extender Kit for Complex *O*-Linked Glycans (GK80115), according to manufacturer’s instructions (Agilent).

### Statistical analysis

The statistical significance of differences was evaluated using Dunnett’s test (Fig. 1, *B*–*E*) and Student’s *t* test with Bonferroni correction (Figs. 3, *C*–*F*, 4, *C*–*F*, 5, *A*–*D*, 6, *B* and *C*, 7, *B*, *D*–*E*, 9, *B*, and *D*), with *p* < 0.05 considered to be statistically significant.

## Data availability

All data are available within the article and supporting information.

## Supporting information

This article contains supporting information (60, 62, 63).

## Conflict of interest

The authors declare that they have no conflicts of interest with the contents of this article.
