# Supplementary material for: Dysregulation of PI4P in the trans Golgi regions activates the mammalian Golgi stress response
Source: J Biol Chem. 2024 Dec 13;301(1):108075. doi: 10.1016/j.jbc.2024.108075 (PMC11770552; doi:10.1016/j.jbc.2024.108075)
Supplement: Supporting Information [file mmc4.docx]

**Supporting Information**

**Figure S1. Immunoelectron microscopy analysis of the Golgi**

After wt HeLa cells and PITPNB KO clones (#2 and #6) were treated with or without 5 nM OSW-1 for 18 h, they were fixed with a mixture of 0.2% gultaraldehyde and 2% paraformaldehyde for 1 h, detached from dishes using a spatula, and embedded in agarose as previously described (Koga et al., 2012). Specimens for immunoelectron microscopy were prepared as previously described (Kusumi et al., 2018). In brief, the agarose containing HeLa cells was cut into small pieces, and these agarose blocks were immersed in a mixture of polyvinylpyrrolidone and high-molar sucrose solution for cryoprotection. They were then mounted on specimen pins, quickly frozen in liquid nitrogen, and placed in a cryochamber attached to an ultramicrotome (Ultracut EM UC7; Leica Microsystems, Nussloch, Germany). Semi-thin cryosections (1 μm thickness) were subsequently cut from the frozen block in the chamber using a diamond knife (Diatome, Biel, Switzerland), picked up with a 2.3 M sucrose droplet on a wire loop, and mounted on glass microscope slides. After the sections were rinsed with phosphate buffered saline (PBS), they were blocked with 5% normal goat serum and incubated with a primary antibody (a mouse monoclonal anti-rat GM130 antibody; BD Biosciences, San Jose, CA) for 12 h at 4°C. Then, the specimens were rinsed with PBS, incubated with a secondary antibody (Alexa Fluor 488-FluoroNanogold-labeled Goat anti-Mouse IgG; Nanoprobes, Yaphank, NY, USA) for 2 h at 20°C, and rinsed again with PBS. The sections on the glass slides were further fixed with 2% glutaraldehyde for 1 h and incubated in a gold enhancing solution (GOLDENHANCETM EM; Nanoprobes) for 3 min at 20°C to visualize the labeled gold particles by scanning electron microscopy. They were subsequently post-fixed with a mixture of 2% osmium tetroxide and 1.5% potassium ferrocyanide for 1 h, dehydrated in a graded ethanol series, embedded in epoxy resin, and polymerized for 48 h at 60°C. After the resin blocks were detached form the glass slides, ultrathin sections (100 nm thick) were cut with the ultramicrotome using a diamond knife (Diatome), attached onto a glass slide by heating on a hot plate, heavy metal stained with uranyl acetate and lead citrate, coated with carbon using a carbon coater (VC-100; Vacuum Device, Ibaraki, Japan), and observed in a semi-in-lens type field emission scanning electron microscope (Regulus; Hitachi, Tokyo, Japan) using the BSE detector with an accelerating voltage at 2 kV. *Solid arrowheads*, gold particles of GM130. Scale bars, 500 nm.

**Supplemental movie 1-4. 3D reconstruction analysis of the Golgi**

After wt HeLa cells and PITPNB KO clone#6 were treated with or without 5 nM OSW-1 for 18 h, the cells were fixed with a mixture of 0.4% gultaraldehyde and 2% paraformaldehyde for 24 h, detached from dishes using a spatula, and embedded in agarose as previously described (Koga et al., 2012). The agarose containing HeLa cells was cut into small pieces and they were prepared according to our paper (Koga et al., 2017). In brief, agarose blocks were post-fixed with 1% osmium tetroxide (0.1 M PB; pH 7.4) for 3 h, rinsed with distilled water and stained with 1% uranyl acetate for 3 h. Subsequently, the blocks were dehydrated using an ascending series of alcohol, embedded in epoxy resin and polymerized for 48 h at 60°C. After trimming the epoxy blocks to the appropriate size, serial ultrathin sections of 100 nm thickness were cut with an ultramicrotome (Ultracut EM UC7; Leica Microsystems, Nussloch, Germany) using a diamond knife (Diatome, Biel, Switzerland). The sections were then attached to glass slides, stained with uranyl acetate and lead citrate for heavy metal staining, coated with carbon using a carbon coater (VC-100; Vacuum Device, Ibaraki, Japan), and observed in an ultra-high-resolution scanning electron microscopes (SU-70 and Regulus, Hitachi, Tokyo, Japan) to acquire sequential images of target cells. After aligning the serial images using a computer software (Amira, Thermo Fisher Scientific, USA), target structures such as the Golgi apparatus, nuclei, and cytoplasm were segmented and reconstructed in three dimensions. The Golgi apparatus, nucleus, vacuoles and cytoplasm are shown in green, blue, magenta and orange, respectively.

Koga D, Nakajima M, and Ushiki T (2012). A useful method for observing intracellular structures of free and cultured cells by scanning electron microscopy. J Electron Microsc: 105-111. doi: 10.1093/jmicro/dfr098.

Koga, D., Kusumi, S., Ushiki, T., and Watanabe, T. (2017). Integrative method for three-dimensional imaging of the entire Golgi apparatus by combining thiamine pyrophosphatase cytochemistry and array tomography using backscattered electron-mode scanning electron microscopy. Biomed. Res. 38, 285–296. doi: 10.2220/biomedres.38.285

Kusumi S., Koga D., Watanabe T, and Shibata M. (2018) Combination of a cryosectioning method and section scanning electron microscopy for immuno-scanning electron microscopy. Biomed. Res. (Tokyo) 39, 21–25. doi: 10.2220/biomedres.39.21.

**Figure S2. OSW-1-induced cell death in HeLa cells expressing EGFP-SAC1 wt and K2A mutant**

(A) Immunofluorescence microscopic analysis of HeLa cells expressing EGFP-fused SAC1 wt and K2A mutant. The cells were fixed in 4% paraformaldehyde in PBS, permeabilized with 0.2% Triton X-100, and then stained with murine anti-GM130 mAb and DAPI. Fluorescence signals in the merged images are represented in pseudo-colors as follows: DAPI, blue; EGFP, green; GM130, red. Scale bars, 50 μm. (B) Cell viability assay. HeLa cells expressing EGFP-fused SAC1 wt and K2A mutant were treated with or without the indicated concentrations of OSW-1 for 48 h, and subjected to cell viability assay. ns, not significant (the Bonferroni-corrected t-test; mean ± S.D.; n = 3).

**Supplemental data 1 and 2. The complete results of RNA-seq.**

Total RNA extracted from HeLa cells treated with or without 5 nM OSW-1 for 18 h was subjected to RNA sequencing analysis. Supplemental data 1 shows the result of gene expression (Reads Per Million reads, RPM) calculated by Torrent Suite software v5.4.0, AmpliSeq RNA plugin (Thermo Fisher Scientific). Supplemental data 2 shows the result of differentially expressed genes analyzed by R. Details of the samples are as follows: IonXpress_001, 003 and 005, the untreated control group; IonXpress_002, 004 and 006, the OSW-1-treated group. The raw and processed data of NGS have been deposited to National Center for Biotechnology Information GEO (accession no. GSE255894).

**Supplemental data 3. The complete result of the CRISPR screening.**

Two sgRNA-expressing HeLa cell libraries (A-1 and A-2) using the GeCKO v2.0 library (Libraries A) were treated with 5 nM OSW-1. Sequencing analysis of genome-integrated sgRNAs in OSW-1-resistant cells and untreated control cells was performed using an Ion Proton System (Thermo Fisher Scientific). Supplemental data 3 shows the results of read count comparison and statistical test using an SgRNA Screening v.1.1 custom plugin (Thermo Fisher Scientific). The meanings of the words are represented as follows: id, sgRNA ID; baseMean, Mean value of normalized read counts among the total samples; baseMeanA, Mean value of normalized read counts in the untreated control group; baseMeanB, Mean value of normalized read counts in the OSW-1-treated group; foldChange, Ratio of read counts (the OSW-1-treated group/ the untreated control group); log2FoldChange, conversion value of read count ratio to log2; pval, p-value calculated by DESeq; padj, p-value calculated by DESeq (Corrected values using the Benjamini-Hochberg method). The raw and processed data of NGS have been deposited to National Center for Biotechnology Information GEO (accession no. GSE255970).
