# Supplementary figures and images for: Dysregulation of PI4P in the trans Golgi regions activates the mammalian Golgi stress response
Source: J Biol Chem. 2024 Dec 13;301(1):108075. doi: 10.1016/j.jbc.2024.108075 (PMC11770552; doi:10.1016/j.jbc.2024.108075)

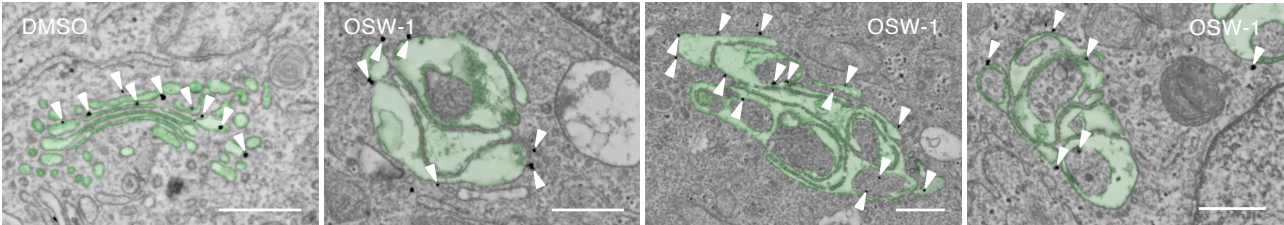

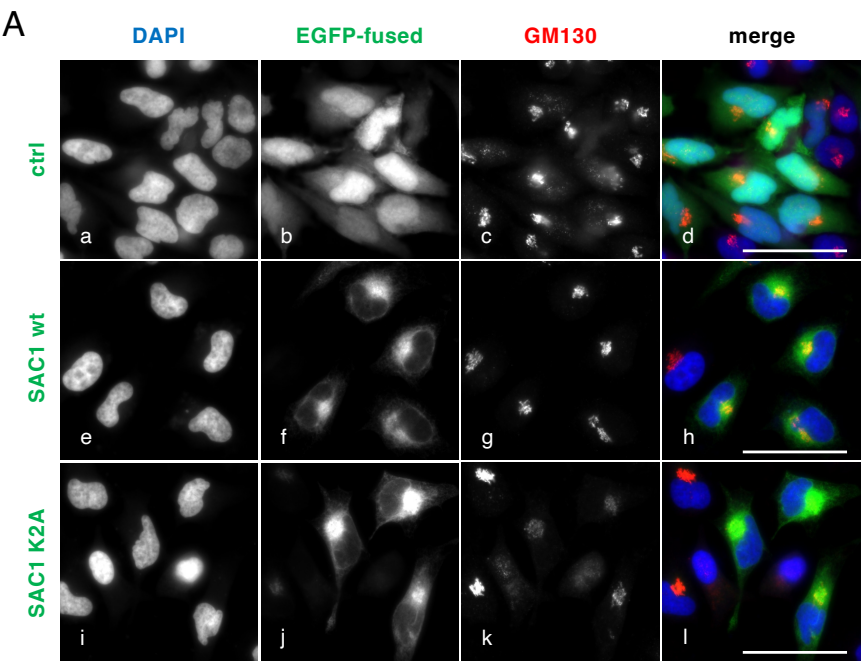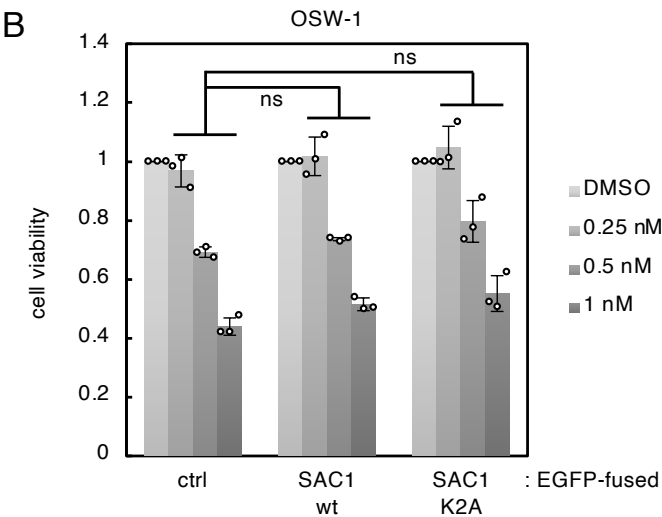

Supplement: Supporting Information_Figure [file mmc5.pdf]
